# Case Report: Whole exome sequencing helps in accurate molecular diagnosis in siblings with a rare co-occurrence of paternally inherited 22q12 duplication and autosomal recessive non-syndromic ichthyosis.

**DOI:** 10.12688/f1000research.6779.1

**Published:** 2015-07-31

**Authors:** Aayush Gupta, Yugal Sharma, Kirti Deo, Shamsudheen Vellarikkal, Rijith Jayarajan, Vishal Dixit, Ankit Verma, Vinod Scaria, Sridhar Sivasubbu

**Affiliations:** 1 Department of Dermatology, Dr. D.Y. Patil Medical College, Pune, India; 2 Genomics and Molecular Medicine, CSIR Institute of Genomics and Integrative Biology, Delhi, India; 3 GN Ramachandran Knowledge Center for Genome Informatics, CSIR Institute of Genomics and Integrative Biology, Delhi, India; 4 Academy of Scientific and Innovative Research, Center for Genome Informatics, CSIR Institute of Genomics and Integrative Biology, Delhi, India

**Keywords:** Whole Exome Sequencing, Lamellar ichthyosis, chr22q12, genodermatosis, TGM1

## Abstract

Lamellar ichthyosis (LI), considered an autosomal recessive monogenic genodermatosis, has an incidence of approximately 1 in 250,000. Usually associated with mutations in the transglutaminase gene ( *TGM1*), mutations in six other genes have, less frequently, been shown to be causative. Two siblings, born in a collodion membrane, presented with fish like scales all over the body. Karyotyping revealed duplication of the chromosome arm on 22q12+ in the father and two siblings. Whole exome sequencing revealed a homozygous p.Gly218Ser variation in *TGM1*; a variation reported earlier in an isolated Finnish population in association with autosomal recessive non-syndromic ichthyosis. This concurrence of a potentially benign 22q12+ duplication and LI, both rare individually, is reported here likely for the first time.

## Introduction

Autosomal recessive congenital ichthyosis (ARCI), a heterogeneous disorder of cornification of skin, encompasses three clinical subtypes: lamellar ichthyosis (LI; OMIM 242300); congenital ichthyosiform erythroderma (CIE; OMIM 242100); and harlequin ichthyosis (HI; OMIM 242500) ^1^. LI has an incidence of approximately 1 in 250,000. Over 115 mutations in *TGM1* and less frequent ones in six other genes ( *NIPAL4z, ALOX12B, CGI-58, FLJ39501, ICHYN* and *ABCA12)* have been associated with the LI/CIE phenotypic spectrum worldwide ^2, 3^. Overlapping phenotypes and the non-specificity of the conventional histopathology, makes clinical diagnosis challenging in many cases and inaccurate in some ^4^. Whole exome sequencing has become a useful diagnostic aid for genetic disorders including multigene dermatoses such as epidermolysis bullosa ^5, 6^ and acrokeratosis verruciformis ^7^.

## Case report

**Figure 1.**
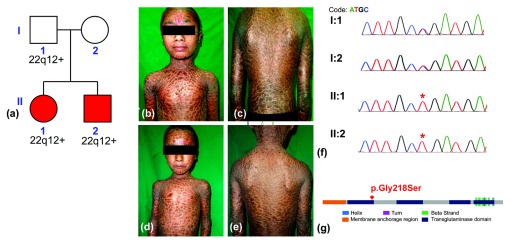
**a**) Pedigree of the family; ( **b**), ( **c**) and ( **d**), ( **e**) correspond to the ventral and dorsal views of siblings II:1, and II:2 respectively and shows hyperpigmented fish-like scales all over the body including face and flexures, ectropion, loss of lateral half of eyebrows and hair along scalp margins. Panel ( **f**) shows the chromatogram from capillary sequencing for the parents and siblings, while panel ( **g**) shows the domain organization of the protein and the location of the p.Gly218Ser variation with respect to the protein domains.

Two, 8 and 6-year-old, siblings born out of a non-consanguineous marriage ( Figure 1a) presented with hyperpigmented fish-like scales all over the body including face and flexures ectropion, loss of lateral half of eyebrows and alopecia along the scalp margins ( Figure 1b–1e). Both siblings were heat intolerant, photosensitive and hypohidrotic. Born uneventfully vaginally they were encased in a collodion membrane which was shed within a week of birth. There was no family history of any dermatoses. Slit lamp examination revealed bilateral keratitis. Karyotyping of their parents and the siblings performed previously revealed duplication of the chromosome arm on 22q12+ in the father and two siblings. The patients were put on daily oral (5 mg) isotretinoin after analysing their lipid profile.

Considering the diagnosis of LI, whole exome sequencing was attempted. Genomic DNA (gDNA) was isolated from 5 ml of blood ^8^ of each of the affected children after obtaining written informed consent conforming to the institutional ethical committee approvals (Dr. D.Y. Patil Vidyapeeth, Pune. Approval number DYPV/EC/178/14 ). The whole exome capture and library preparation (Nextera expanded exome, Illumina Inc., USA) were carried out according to the manufacturer’s instructions and followed by high throughput sequence generation on Hiseq 2500 with default 101 paired end single index sequence by synthesis chemistry (Illumina Inc., USA). The raw sequence reads were trimmed at a Phred score of 30 leaving over 44.9 and 33.45 million reads respectively for the two siblings. The variations were called against the hg19 version of the human genome using standard GATK-Picard pipeline with Burrows-Wheeler Alignment according to GATK best practice ^9^. The variants from the genomes of both siblings were further analysed using ANNOVAR ^10^ for coding region and also screened using the NCBI-Clinvar database ( http://www.ncbi.nlm.nih.gov/clinvar/). Analysis revealed a homozygous p.Gly218Ser variation in *TGM1* previously reported to be associated with autosomal recessive non-syndromic ichthyosis in an isolated Finnish population ^11^. The variant mapped to the transglutaminase domain in the protein ( Figure 1g) and was also predicted to be pathogenic by both SIFT (Sorts Intolerant From Tolerant) ^15^ and PolyPhen2 ^16^ (Polymorphism Phenotyping v2). This variation was further validated in parents and both siblings by site-specific PCR using a forward primer CTTCTCCTGGGGTCAGGCA and reverse primer GAGAAGTCCCAGGCTCCATC (Sigma Aldrich). The PCR was done using *taq* polymerase (Invitrogen, USA, Cat. No. 10342053) according to the manufacturer supplied protocol with a Tm of 60.5°C. The PCR products were size selected and gel purified (2% agarose) using qiaquick gel extraction kit (QIAGEN, NL) and performed capillary sequencing (Applied Biosystems) performed using manufacturer instruction. Analysis revealed the variant was heterozygous in parents, while homozygous in both affected siblings ( Figure 1f).

Follow up after two years of low dose isotretinoin, titrated intermittently, revealed complete subsidence of ectropion, eclabium and alopecia with residual fine scales.

## Discussion

ARCI is a rare disorder with an estimated prevalence of 1 per 200,000 population in Europe and 1 per 200,000–300,000 population in the United States. Neonates with LI typically present with a collodion membrane which dries and peels away and is replaced by brown, plate-like scales over the entire body. Disease course ranges from very mild to severe, latter entailing ectropion, eclabium, scarring supraciliary and scalp alopecia, and palmoplantar hyperkeratosis ^11^.

DNA based molecular diagnosis is crucial in ichthyosis as it provides a firm basis for genetic counseling of affected individuals and families, and also permits prenatal diagnosis. In a cohort of 520 independent families with ARCI, mutations were identified by direct sequencing of the 6 ARCI genes identified to date in 78% of patients: 32% harbored mutations in *TGM1*, 16% in *NIPAL4*, 12% in *ALOX12B*, 8% in *CYP4F22*, 5% in *ABCA12*, and 5% in *ALOXE3*. Whole exome sequencing may fill in the diagnostic lacuna of at least 22% of the patients who failed in this study to exhibit mutations in any of the known ARCI genes, indicating the existence of additional loci, such as 2 loci on chromosome 12p11.2-q13 ^12^. The 22q12+ duplication is known to cause cat eye syndrome, which has a range of potential morbidities with the occurrence of characteristic triad of iris coloboma, aural tags and/or pits and anal atresia ^14^, though none of these features were present in the father or the children.

To the best of our knowledge, this is the first reported concurrence of a potentially benign 22q12+ duplication and LI, both of which are extremely rare individually. The mother of the siblings is now pregnant and the present finding will be used to help screen the foetus prenatally.

## Consent

Written informed consent for publication of their clinical details and clinical images was obtained from the parent of the patients.

## Data availability

The raw exome sequencing data are available at the NCBI Sequence Read Archive ( http://www.ncbi.nlm.nih.gov/sra), accession numbers SRX1096915 (II:1) and SRX1096920 (II:2). 
