# Supplementary material for: Case Report: Whole exome sequencing helps in accurate molecular diagnosis in siblings with a rare co-occurrence of paternally inherited 22q12 duplication and autosomal recessive non-syndromic ichthyosis
Source: F1000Res. 2015 Jul 31;4:446. [Version 1] doi: 10.12688/f1000research.6779.1 (PMC4648218; doi:10.12688/f1000research.6779.1)
Supplement: Peer review history

# Peer review: Referee response for version 1

**DOI:** 10.5256/f1000research.7283.r10062

**Published:** 2015-08-24

**Authors:** Regina Fölster-Holst

**Affiliations:** 1 Department of Dermatology, Allergology and Venerology, University of Kiel, Kiel, Germany

Gupta *et al. *focused on two siblings with ARCI with confirmed TGM1 mutation. In addition they found 22q12 duplication in these siblings and in their father. The title and abstract are both appropriate for the article, and the abstract is a suitable summary. The diagnosis has been sufficiently described, however it is a little bit unclear whether the 22q12 duplication is associated with the ARCI. The authors mention the cat eye syndrome which is related to the duplication of the chromosome 22q12. What about the relevance of this finding? It should be worked out in more detail.

I have read this submission. I believe that I have an appropriate level of expertise to confirm that it is of an acceptable scientific standard.

# Peer review: Referee response for version 1

**DOI:** 10.5256/f1000research.7283.r9750

**Published:** 2015-08-10

**Authors:** Mohamed Badawy Abdel-Naser

**Affiliations:** 1 Faculty of Medicine, Ain Shams University , Cairo, Egypt

- The authors reported 2 siblings with lamellar ichthyosis (LI) and the family pedigree shows the autosomal pattern of inheritance. In addition, duplicate 22q12+ has been shown in the father and the 2 affected siblings. **It seems, however, that this chromosomal abnormality is unrelated to the LI **as the father is phenotypically normal and the 2 siblings do not show any of the manifestations of this chromosomal abnormality. **The significance of this concurrence is not clear. **
- The authors may amend the manuscript by naming the site of the TGM1 mutation at the nucleotide level and the name of the gene transcript.
- It is not clear whether the authors examined the father and the affected siblings for manifestations of the duplicate 22q12+ (apart from the cat eye syndrome), such as learning difficulties, growth retardation, minor genital abnormalities of the boy etc.
- There are few typo errors. For instance, the "transglutaminase gene" is better given as "transglutaminase gene1" and "Ichyn" is "Ichthyin" etc.
- Ref.13 is not cited in the text and there seems to be a problem with enumeration of the references in the text. ​

I have read this submission. I believe that I have an appropriate level of expertise to confirm that it is of an acceptable scientific standard.
